# Ingratitude

**Published:** 1887-07

**Authors:** 


### INGRATITUDE.

A true and complete education may be classed under three divisions—mental, physical, and emotional. Though all are of great importance, the latter is of the most value. The cultivation of the emotions is repressing the bad and cherishing the good, and this constitutes man's moral education. Emotion is the expression of feeling by words, acts or signs, and from these feelings all our duties result. Without this capacity our intellectual nature would be cold, valueless and dead, and life would be one bleak and monotinous field of action. Of all emotions to which our fallen nature is prone, scarcely any is more base than Ingratitude. It is demoralizing to the heart and conscience of man, and to cherish it in words or acts is to cherish a spirit of evil that will destroy the virtues which struggle to get possession of a man's soul. Selfishness is another effective agent in marring the beauty and symmetry of one's character, and preventing his usefulness. Nothing can be more unlovable and unattractive; and it is a singular fact that a selfish person is also an ungrateful one, and is prone to self-conceit and self-glorification. Such a man can never make happy those about him, or can ever be happy himself. He cannot be a blessing to his fellow men, nor can he honorably attain an honorable position among them.

In no soundings of the human heart has Shakspeare more evinced his knowledge of its every emotion than where he says:

"How sharper than a serpent's tooth
It is to have a thankless child."

To this one may add, how sharp is the pain to have an ungrateful friend, associate, or beneficiary of any kind, to whom one has revealed the best thoughts of his mind—who has freely given the precepts and example of a noble life, tendered wise counsel and affectionate words of exhortation, and extended the helping hand on every occasion when it was possible to give the friendly response.

To young Doctors of Medicine I would earnestly say, Cultivate the beautiful and ennobling virtue of Gratitude. Without it, you cannot be loved and esteemed; you cannot be honored in the hearts of confreres, and receive their approval of your private and professional character. In these days of lightning speed, do not be carried away by the current of Self-pride; do not think, my young friends, that you "know it all," and that, in the race, you are far ahead of the honored veterans of the profession, or "old fogy doctors" as some call them. The "old fogies" are always glad to welcome any revelation in medical science which will "lessen the ills that flesh is heir to," and they rejoice to see the profession moving up to the vanguard of the times; but it should be remembered that everything has a beginning—that the foundation of every science was laid by some bold hand and fearless spirit, and that the pioneer in any movement has the undying honor of opening the way for those who may follow.

We do not write the foregoing from any personal motives, nor in a spirit of censure towards any of our young professional brethren, but simply from a great interest in all that pertains to the highest welfare of those who, in the future, will take the places of their predecessors, and who, in both private and professional character, we would have to honor the noble calling which the writer so loves, and to which he has given the best years of his life.

Wisdom is attained by putting knowledge to a series of tests that will refute or establish the truth of what one has learned. Therefore, it is not alone the experience of years which makes one wise, but it is also the improvement of opportunities, and the most intelligent use of the means provided. However there are some physicians of comparatively few years' of practical experience who are better informed than some of long service in the profession, because the latter have sought and grasped but few opportunities to increase in progressive information. But a physician who, to much and varied experimental knowledge, adds the wisdom of accumulated years, the virtues of a noble, true-hearted man, and by his honorable and honored life has exalted the animus of the profession, such a physician has no feeling of envy or jealousy towards the young doctor whom he sees giving promise of taking high rank in the profession and becoming a rich blessing to suffering humanity, yet is so modest and unassuming in this successful emulation, so respectful towards his elders, and considerate of the honest opinions of others, he commands both respect and esteem by the very force of his noble personality. On the contrary, when this old doctor looks upon young physicians of the above character, his heart throbs with professional pride, for he sees in him a worthy representative of that noble calling to which he has given the best years of his life, and desired to have honored above all others of an earthly character. In him the young professional brother will always find a noble mentor, a safe guide, and a true, unselfish friend.

